# A branching morphogenesis program governs embryonic growth of the thyroid gland

**DOI:** 10.1242/dev.146829

**Published:** 2018-01-15

**Authors:** Shawn Liang, Ellen Johansson, Guillermo Barila, Daniel L. Altschuler, Henrik Fagman, Mikael Nilsson

**Affiliations:** 1 Sahlgrenska Cancer Center, Institute of Biomedicine, Department of Medical Chemistry and Cell Biology, University of Gothenburg, SE-40530, Göteborg, Sweden; 2 Department of Pharmacology and Chemical Biology, University of Pittsburgh School of Medicine, Pittsburgh, PA 15261, USA; 3 Department of Clinical Pathology and Genetics, Sahlgrenska University Hospital, SE-41345, Göteborg, Sweden

**Keywords:** Thyroid, Progenitor, Fgf10, Sox9, Mouse, Growth, Differentiation

## Abstract

The developmental program that regulates thyroid progenitor cell proliferation is largely unknown. Here, we show that branching-like morphogenesis is a driving force to attain final size of the embryonic thyroid gland in mice. Sox9, a key factor in branching organ development, distinguishes Nkx2-1^+^ cells in the thyroid bud from the progenitors that originally form the thyroid placode in anterior endoderm. As lobes develop the thyroid primordial tissue branches several generations. Sox9 and Fgfr2b are co-expressed distally in the branching epithelium prior to folliculogenesis. The thyroid in *Fgf10* null mutants has a normal shape but is severely hypoplastic. Absence of *Fgf10* leads to defective branching and disorganized angiofollicular units although Sox9/Fgfr2b expression and the ability of cells to differentiate and form nascent follicles are not impaired. These findings demonstrate a novel mechanism of thyroid development reminiscent of the Fgf10-Sox9 program that characterizes organogenesis in classical branching organs, and provide clues to aid understanding of how the endocrine thyroid gland once evolved from an exocrine ancestor present in the invertebrate endostyle.

## Abstract (precis)

**Summary:** Embryonic mouse thyroid gland develops by branching morphogenesis stimulated by mesenchymal Fgf10 targeting Sox9^+^/Nkx2-1^+^ progenitors, in a mechanism reminiscent of ductal organ development and thyroid evolution from an exocrine forerunner.

## INTRODUCTION

Mouse thyroid morphogenesis recapitulates in less than one week the developmental program of the human thyroid gland (Nilsson and Fagman, 2017). In this process, progenitors of the follicular cell lineage undergo several distinct phases of growth. Cells in anterior endoderm are induced to a thyroid fate by the concerted action of fibroblast growth factor 2 (Fgf2) and bone morphogenetic protein 4 (Bmp4) (Kurmann et al., 2015), both of which probably derive from cardiogenic mesoderm (Serls et al., 2005; Wendl et al., 2007). Together with a Tbx1/Fgf8-dependent signal from mesoderm (Lania et al., 2009), these factors promote the generation of thyroid progenitors, which assemble in the midline of the pharyngeal floor from which the thyroid bud or diverticulum develops. Notably, bud enlargement is not accomplished by localized proliferation indicating that embryonic thyroid growth at this developmental stage is achieved by annexing cells from adjacent endoderm (Kinebrew and Hilfer, 2001; Fagman et al., 2006). It is not until the detached thyroid primordium migrates downwards that progenitor cells are triggered to multiply intensely leading to the prospective gland, after finishing migration, bifurcating and growing bilaterally (Fagman et al., 2006); this represents the starting point of the bilobation process. A third distinct growth pattern appears after the lateral thyroid lobes are formed by fusion with the paired ultimobranchial bodies (UBBs), the latter of which provide C cell precursors to the embryonic thyroid (Johansson et al., 2015). This growth phase involves a subpopulation of rapidly multiplying progenitors that occupy the peripheral or distal parts of the parenchyma as the lobe enlarges (Fagman et al., 2006). However, molecular mechanisms that govern primordial growth of the embryonic thyroid beyond the bud stage are poorly understood (Nilsson and Fagman, 2017).

Transcriptional profiling of mouse primordial tissues *in vivo* (Fagman et al., 2011) and recent *in vitro*-directed differentiation of human pluripotent stem cells (Serra et al., 2017) reveal distinct developmental traits of thyroid and lung organogenesis. However, it is evident that thyroid and lung progenitor cells share regulatory pathways most notably distinguished by the homeobox transcription factor Nkx2-1, which is required for both organs to develop normally from foregut endoderm (Kimura et al., 1996). Accordingly, in *Nkx2-1*-deficient mice thyroid development is arrested at the bud stage and the primordium degenerates (Parlato et al., 2004), probably as a result of diminished Bcl2 expression (Fagman et al., 2011; Porreca et al., 2012), whereas the lungs show impaired distal branching leading to severe hypoplasia of the bronchial tree and defective alveolarization (Minoo et al., 1999; Yuan et al., 2000). Analogous to the role of the phosphorylated form of Nkx2-1 in late lung development (DeFelice et al., 2003), phospho-Nkx2-1 is required for follicle formation constituting the final stage of thyroid morphogenesis (Silberschmidt et al., 2011). On the other hand, the transcriptional networks in which Nkx2-1 participates are differentially regulated and likely confer lineage specificity in thyroid and lung (Parlato et al., 2004; Rankin and Zorn, 2014). The extent to which Nkx2-1^+^ thyroid and lung progenitors share morphogenetic mechanisms with possible impact on growth control is presently unknown.

Recent studies indicate that differentiation of embryonic or induced pluripotent stem cells into a distal Nkx2-1^+^/Sox9^+^ lung phenotype requires the presence of embryonic lung mesenchyme, and that the effect is likely mediated by Fgf10-Fgfr2b signaling (Fox et al., 2015). This is consistent with the documented crucial role *in vivo* of Fgf10, governed by reciprocal mesenchymal-epithelial interactions, in lung branching morphogenesis (Bellusci et al., 1997; Sekine et al., 1999; Abler et al., 2009). In this process, Fgf10 stimulates Nkx2-1^+^ lung progenitor cell proliferation (Ramasamy et al., 2007) and survival (Abler et al., 2009), and preserves the distal, immature lung phenotype (Volckaert et al., 2013). Moreover, Sox9 acts downstream of Fgf coordinating cell-autonomously the branching program in the developing lung (Chang et al., 2013; Rockich et al., 2013). In the present study on the embryonic mouse thyroid, we find that Sox9 is expressed in a subpopulation of distal progenitors during budding and lobular growth prior to functional differentiation. We further demonstrate that proliferation of Nkx2-1^+^/Sox9^+^ thyroid progenitors requires Fgf10, derived from surrounding mesenchyme, which stimulates epithelial branching growth. This uncovers a novel mechanism of thyroid development reminiscent of branching morphogenesis that otherwise is a hallmark of ductal and exocrine organs.

## RESULTS

### Sox9 distinguishes placode from bud progenitors in early thyroid development

**Fig. 1.**
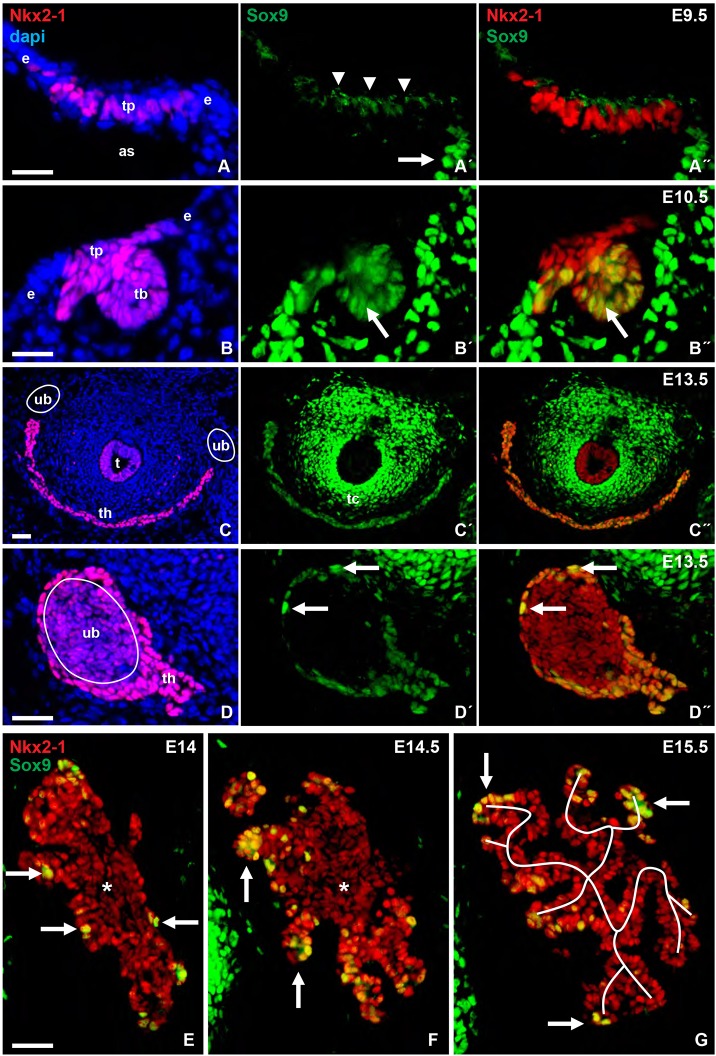
**Sox9 expression in mouse thyroid development.** Thyroid progenitors were identified by Nkx2-1 expression. (A-A″) Thyroid placode. Arrow and arrowheads indicate nuclear and cytoplasmic Sox9, respectively. (B-B″) Thyroid bud. Arrows indicate Nkx2-1/Sox9 colocalization. (C-C″) Thyroid primordium, isthmus portion. (D-D″) Thyroid primordium enclosing the ultimobranchial body (encircled). Arrows indicate Sox9^high^ thyroid progenitors. (E-G) Branching growth pattern of prospective thyroid lobe (branches outlined in G). Arrows indicate Nkx2-1^+^/Sox9^high^ cells; asterisks indicate residual ultimobranchial body. as, aortic sac (positioned close to thyroid placode); dapi, nuclear stain; e, endoderm; t, trachea; tb, thyroid bud; th, thyroid primordium; tp, thyroid placode; ub, ultimobranchial body (position encircled). Scale bars: 25 µm (A,B); 100 µm (C); 50 µm (D-G).

Thyroid progenitors do not prematurely differentiate although the transcriptional factors necessary for functional differentiation are expressed already in early development as the thyroid lineage is specified in anterior endoderm (Nilsson and Fagman, 2017). The mechanisms that prevent thyroid differentiation until cells have formed follicles in late organogenesis are unknown. Sox9 has gained increasing interest for its transcriptional role in maintaining the progenitor state of cells in organ development (Jo et al., 2014). This led us to investigate whether Sox9 is expressed in the follicular cell lineage. In the thyroid placode (Fig. 1A), Nkx2-1^+^ progenitors started to express Sox9 in the cytoplasm at embryonic day (E) 9.5 (Fig. 1A′,A″). One day later, Sox9 showed nuclear accumulation in the bud but not in Nkx2-1^+^ cells retained in the placode (Fig. 1B-B″). Sox9 also distinguished distal from proximal Nkx2-1^+^ cells as the bud invaginated and detached from the endoderm (Fig. S1A-C). A similar Sox9 pattern was observed in the Nkx2-1^+^ lung bud (Fig. S1E-E‴) whereas the UBBs starting to express Nkx2-1 were Sox9 negative (Fig. S1D). These dynamic changes of Sox9 expression during thyroid budding conforms with previous notions that key transcription factors are differentially regulated in thyroid progenitors transiting from the placode to the bud stage (Parlato et al., 2004). Notably, Sox9^+^ mesenchyme accumulated near the thyroid primordium (Fig. 1B′,B″, Fig. S1A-C) and the developing UBBs (Fig. S1D) but did not appear in the branching lung bud (Fig. S1E-E‴), suggesting that Sox9 might regulate early thyroid development also non-cell-autonomously.

### Differential expression of Sox9 in distal progenitors reveals a branching pattern of thyroid morphogenesis

In the next developmental stages, the thyroid primordium migrates downwards followed by its bifurcation, which constitutes the starting point of the bilobation process. Upon thyroid fusion with the UBBs, thyroid progenitors were in general weakly Sox9 positive (Sox9^low^) with the exception of a minority of cells that showed strong Sox9 expression (Sox9^high^) and that were exclusively present, either singly or in small clusters, on the UBB surface (Fig. 1C-D″). In the following days, corresponding to pre-follicular growth, the prospective thyroid lobes assumed a conspicuous branching pattern of growth by which epithelial cords radiated from the UBB remnant. Notably, in this process Sox9^high^ cells were not randomly distributed but accumulated peripherally (Fig. 1E-G), thus resembling the expression pattern of Sox9 in classical branching organs (Thomsen et al., 2008; Chang et al., 2013; Rockich et al., 2013; Chatzeli et al., 2017). Moreover, although occasional Sox9^high^ cells were encountered centrally (Fig. 1F,G) most parenchymal cells present in this part of the lobe showed low or no Sox9 expression, suggestive of gradual downregulation of Sox9 as cells attained a more proximal position (Fig. S2). This differs from bronchial tree development in which Sox9 is more strictly expressed in the distal termini (Chang et al., 2013). It was also evident that thyroid branching growth did not follow the highly ordered dichotomous branching typical for the developing lung (Chang et al., 2013) but was instead reminiscent of the pseudoglandular stage of salivary gland development before generation of ducts (Chatzeli et al., 2017). These observations uncover a novel feature of thyroid development by which a branching morphogenesis program conceivably drives parenchymal growth and the formation of rudimentary lateral lobes before progenitors differentiate into follicular cells.

### Sox9 expression persists in differentiated thyroid follicular cells

**Fig. 2.**
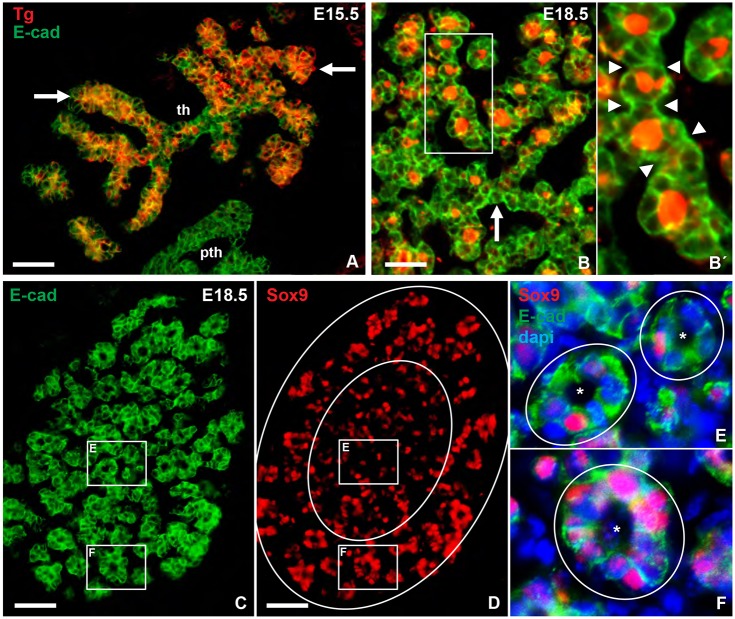
**Sox9 expression during thyroid differentiation and folliculogenesis.** (A) Onset of thyroglobulin (Tg) expression. Arrows indicate distal branches. (B,B′) Distribution of thyroid follicles; B′ shows magnification of the boxed area in B. Arrowheads indicate connections between nascent follicles. Arrow indicates pre-follicular parenchyma. (C,D) Distribution of follicles (C) and Sox9^+^ cells (D); large circles in D distinguish central and peripheral tissues. (E,F) Magnifications of the boxed areas in C and D, as indicated, with the follicles and lumen marked. pth, parathyroid; th, thyroid. Scale bars: 50 µm (A,C,D); 25 µm (B).

Thyroid parenchyma branched one to two generations before the majority of cells started to express thyroglobulin (Fig. 2A). As cells further differentiated to form genuine follicles with the lumens filled with thyroglobulin (Fig. 2B) those follicles appeared back-to-back, unseparated, in contiguous epithelial strands (Fig. 2B′). Interestingly, folliculogenesis occurred synchronously with no apparent preference for the central or peripheral parts of the lobes (Fig. 2C), which contrasted with the peripheral predominance of Sox9^high^ cells (Fig. 2D). As a result, the relative contribution of cells with strong Sox9 expression varied considerably between newly formed follicles (Fig. 2E,F). Together, these observations indicated that thyroid differentiation and folliculogenesis are not restricted to Sox9 negative progenitors, which differs from lung development in which differentiation is pre-patterned by the exclusive proximal-distal expression of Sox2 and Sox9 (Gontan et al., 2008; Chang et al., 2013).

In the adult thyroid, the majority of follicular cells expressed Sox9 (Fig. S3A,B). It was previously reported that Sox9 is downregulated during pancreatic endocrine differentiation (Seymour et al., 2007), and we observed here that Sox9 was lost in the UBBs and parathyroid before these primordia approached the thyroid (Fig. 1D′,D″, Fig. S4A,B). The thyroid thus differs from other endocrine derivatives of foregut endoderm by expressing Sox9 in fully differentiated cells. Notably, many follicular cells showed reciprocal expression levels of Nkx2-1 and Sox9 (Nkx2-1^high^/Sox9^low^ or Nkx2-1^low^/Sox9^high^), the occurrence of which varied among follicles (Fig. S3C).

### Sox9 identifies a subset of proliferating thyroid progenitors

**Fig. 3.**
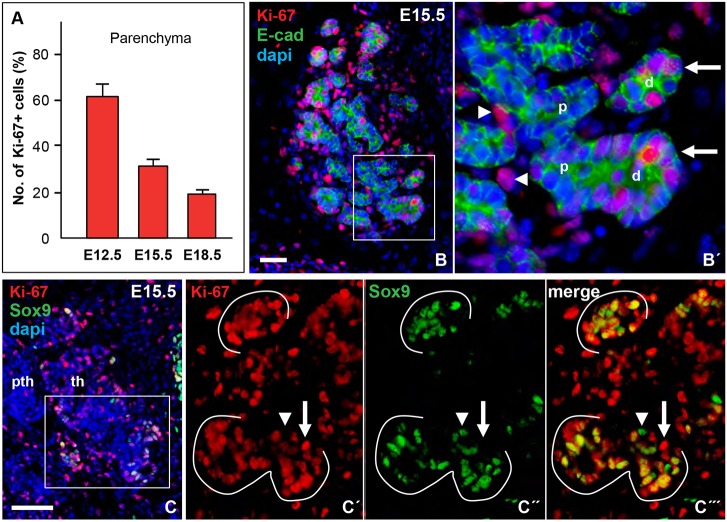
**Progenitor cell proliferation in embryonic thyroid.** (A) Percentage of Ki-67^+^/E-cadherin^+^ thyroid cells (mean±s.d.) at E12.5 (*n*=6), E15.5 (*n*=4) and E18.5 (*n*=4). (B,B′) Tissue distribution of Ki-67^+^ cells in prospective thyroid lobe; B′ shows magnification of the boxed area in B showing distal (d) and proximal (p) cord termini. Arrows and arrowheads indicate epithelial and mesenchymal cells, respectively. (C-C‴) Co-expression of Ki-67 and Sox9; C′-C‴ show single channels of the boxed area in C (rotated 90°). Arrows and arrowheads indicate Ki-67^+^/Sox9^–^ and Ki-67^–/^Sox9^+^ cells (branch tips outlined). dapi, nuclear stain; pth, parathyroid; th, thyroid. Scale bars: 25 µm (B); 50 µm (C).

Because Sox9 regulates branching morphogenesis by preserving a pool of undifferentiated and growth-prone progenitors in the lung (Chang et al., 2013; Rockich et al., 2013) and exocrine glands (Seymour et al., 2012; Chen et al., 2014; Chatzeli et al., 2017), we investigated the possibility that it has a similar role in thyroid development by first monitoring the distribution of Ki-67 (Mki67)-expressing cells during branching growth of the embryonic thyroid. At this stage, Ki-67^+^ cells comprised approximately one-third of the total epithelial cell number (Fig. 3A). For comparison, the proliferation rate was twice as high before fusion with the UBB as the thyroid primordium extended bilaterally, and was further decreased in late glandular development accompanying formation and maturation of follicles (Fig. 3A). This indicated that the relative number of cycling cells gradually decreased. Notably, Ki-67^+^ cells were enriched in the distal tips of epithelial cords whereas proliferating mesenchymal cells were present also between branches inside the lobe (Fig. 3B,B′). Most distal Ki-67^+^ epithelial cells co-expressed Sox9 (Fig. 3C-C‴). However, Ki-67^+^/Sox9^–^ and Ki-67^–^/Sox9^+^ cells were also encountered in branching tips (Fig. 3C-C‴). Ki-67^+^/Sox9^–^ cells were additionally present in more central parts of the lobe. This suggested that at least two populations of cycling epithelial progenitors, distinguished by their tissue distribution and whether Sox9 is expressed or repressed, co-exist in the developing thyroid.

We investigated *Nkx2-1Cre;Sox9^fl/fl^* mice for a possible thyroid phenotype. Inactivation of *Sox9* in the embryonic thyroid was evident although distal Sox9^high^ cells were still encountered in mutants (Fig. S5A). However, this did not significantly alter the branching pattern of thyroid growth (Fig. S5A), and similar results were obtained for the developing lung (Fig. S5B). Recombining *Nkx2-1Cre* with a double fluorescent reporter (*mTmG*) revealed considerable mosaicism at critical stages of morphogenesis (Fig. S5C), which was evident also postnatally (Fig. S5D). Sox9 inactivation with a stronger *Cre* driver such as *Shh*, which was previously reported to generate a Sox9-deficient lung phenotype (Chang et al., 2013), was not possible because thyroid progenitors derive from a Shh-negative lineage of anterior endoderm (Westerlund et al., 2013).

### Epithelial-mesenchymal expression of Fgfr2b and Fgf10 in embryonic thyroid

**Fig. 4.**
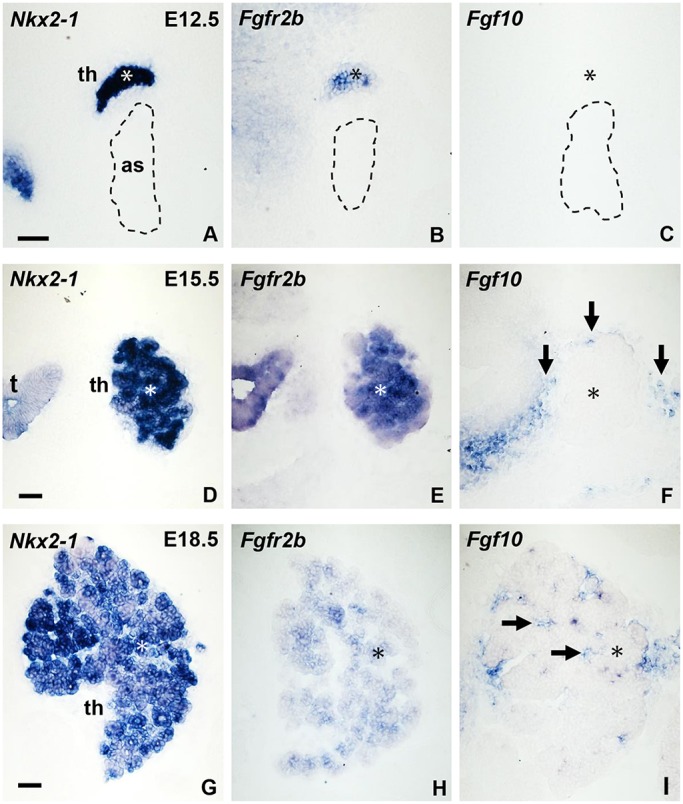
**Expression of *Nkx2-1*, *Fgfr2b* and *Fgf10* in mouse thyroid development.** (A-I) ISH of *Nkx2-1* (A,D,G), *Fgfr2b* (B,E,H) and *Fgf10* (C,F,I). Asterisks indicate location of thyroid. as, aortic sac (encircled); t, trachea; th, thyroid. Arrows indicate *Fgf10*^+^ stroma. Scale bars: 50 µm.

Our findings of branching growth of the embryonic thyroid led us to investigate the possible involvement of Fgf10, which is the most prominent driver of branching morphogenesis and also regulates the expression and function of Sox9 in the developing lung and exocrine glands (Seymour et al., 2012; Chang et al., 2013; Chen et al., 2014; Chatzeli et al., 2017). First, the transcriptional expression of *Fgf10* and its cognate receptor *Fgfr2b* were studied by *in situ* hybridization (ISH) in parallel with that of *Nkx2-1* to distinguish thyroid epithelial progenitors from investing mesenchyme. As shown in Fig. 4A-C, at E12.5 the thyroid primordium weakly expressed *Fgfr2b* whereas there were no detectable *Fgf10* transcripts in or surrounding it. At E15.5, the *Fgfr2b* signal was abundant in the prospective lobe whereas a weak but distinct zone of *Fgf10*-positive mesenchyme was noticed along the outer border of the lobe (Fig. 4D-F). The *Fgf10* expression pattern conformed to the distribution of neural crest-derived cells investing the thyroid (Fig. S6). At E18.5, *Fgfr2b* expression in the thyroid was weaker and more widespread than 3 days before, and *Fgf10* transcript was also detected inside the lobe (Fig. 4G-I).

**Fig. 5.**
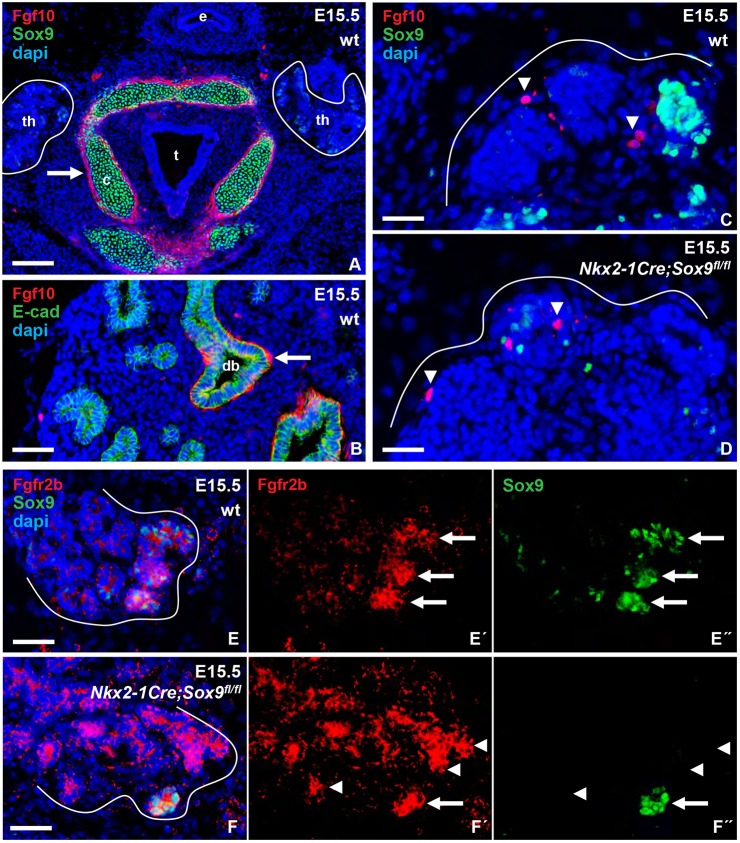
**Fgf10 and Fgfr2b expression in embryonic thyroid wild type and after Sox9 targeted knockout.** Outer border of thyroid primordium is outlined for clarity. (A-D) Fgf10 in larynx (A; thyroid outlined), lung (B), and thyroid (C,D). (E-F″) Fgfr2b in thyroid. Arrows indicate Fgf10^+^ tissues (in A,B) and Fgfr2b^+^/Sox9^+^ cells (in E′,E″,F′,F″), arrowheads indicate Fgf10^+^ stromal cells (in C,D) and Fgfr2b^+^/Sox9^−^ cells (in E′,E″,F′,F″). c, cartilage; dapi, nuclear stain; db, distal bronchus; e, esophagus; t, trachea; th, thyroid. Scale bars: 100 µm (A); 50 µm (B,E,F); 25 µm (C,D).

The mRNA expression pattern suggested that stromal cells invading the embryonic thyroid during lobe formation produced Fgf10. This was confirmed with an Fgf10 antibody that readily stained mesenchyme around Sox9^+^ laryngeal cartilage (Fig. 5A) and distal lung airways (Fig. 5B). However, the number of Fgf10^+^ cells in the thyroid during branching growth was fairly low (Fig. 5C) and did not change in *Nkx2-1Cre;Sox9^fl/fl^* mice (Fig. 5D). Nonetheless, those Fgf10^+^ cells were almost exclusively found close to distal branch endings in the peripheral zone of the lobe. This is of particular interest because Fgfr2b immunoreactivity was much stronger in distal epithelial cells co-expressing Sox9 than in other parts of the gland in which Sox9 was poorly or not expressed (Fig. 5E-E″). Previous studies have obtained diverging results on whether Sox9 promotes the expression of Fgfr2b and hence Fgf10-stimulated branching morphogenesis (Seymour et al., 2012; Chen et al., 2014). As Sox9 was partially lost in the mosaic thyroid of *Nkx2-1Cre;Sox9^fl/fl^* mice, we used this model to investigate whether Sox9 might regulate Fgfr2b in thyroid progenitors. As shown in Fig. 5F-F″, although Fgfr2b had a more widespread distribution in mutant thyroids the receptor was still enriched in branching tips devoid of Sox9^+^ cells (Fig. 5F). Together, these findings indicated that Fgfr2b and Sox9 are co-expressed in distal thyroid progenitors but probably independently regulated.

### Fgf10 inactivation leads to symmetric hypoplasia of the bilobed thyroid gland

**Fig. 6.**
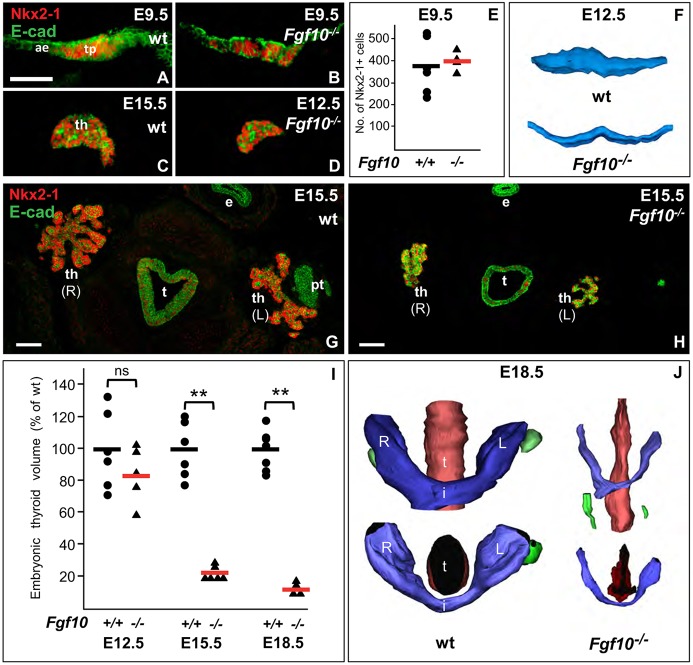
**Thyroid hypoplasia in *Fgf10*-deficient mice.** (A,B) Thyroid placode. (C,D) Thyroid primordium (th), sagittal section. (E) Number of Nkx2-1^+^ thyroid progenitors; wt, *n*=6; knockout (ko), *n*=4. (F) Thyroid primordium, 3D reconstruction. (G,H) Thyroid gland (th), isthmus portion missing in images. (I) Thyroid volume (mean values indicated with black/red lines) at E12.5 (wt, *n*=6; ko, *n*=5), E15.5 (wt, *n*=6; ko, *n*=6) and E18.5 (wt, *n*=7; ko, *n*=4). **P<0.001; ns, not significant. (J) Thyroid gland (blue), 3D reconstruction. Upper panel, frontal view; lower panel, cranial view. ae, anterior endoderm; e, esophagus; i, isthmus; L, left lobe; pt, parathyroid (green); R, right lobe; t, trachea (red); tp, thyroid placode. Scale bars: 25 µm (A,B); 50 µm (G,H).

**Fig. 7.**
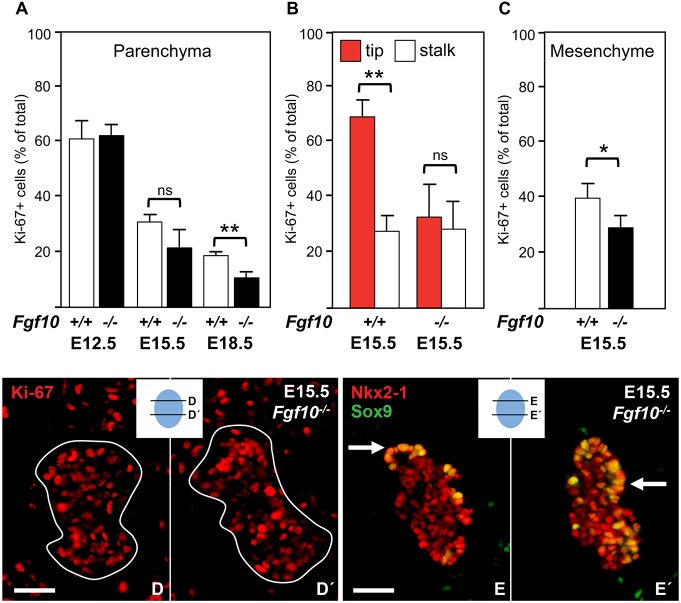
**Proliferation of Sox9^+^ and Sox9^–^ progenitors in *Fgf10*-deficient thyroid.** (A-C) Percentage of Ki-67^+^ cells (mean±s.d.) in thyroid parenchyma (A), tip versus stalk termini (B) and thyroid stroma (C) at E12.5 (wt, *n*=6; ko, *n*=4), E15.5 (wt, *n*=4; ko, *n*=5) and E18.5 (wt, *n*=4; ko, *n*=6). **P<0.001 (in A,B) and *P<0.05 (in C); ns, not significant. (D-E′) Distribution of Ki-67^+^ cells (D) and Sox9^+^ cells (E); D′and E′ show other sections of the same specimens (as indicated in the schematics). Arrows indicate Sox9^+^ cell clusters. Scale bars: 25 µm (D-E′).

Consistent with a recent study (Teshima et al., 2016), the expression pattern of Fgfr2b and Fgf10 in the embryonic thyroid indicated that the Fgf10-Fgfr2b signaling pathway likely regulates thyroid organogenesis rather than being required for thyroid specification and early thyroid development, as suggested from previous observations of thyroid agenesis in *Fgf10*-deficient mice (Ohuchi et al., 2000). We therefore reinvestigated the *Fgf10* null mutant in more detail to characterize further the thyroid defect. In early thyroid development (E9.5-E12.5), no significant alterations were observed between Fgf10^+/+^ and Fgf10^−/−^ embryos regarding size of primordium (Fig. 6A-D,F,I), number of Nkx2-1^+^ progenitors (Fig. 6A,B,E) and fraction of Ki-67^+^ cells (Fig. 7A). However, thyroid lobe enlargement was significantly retarded in *Fgf10* null embryos, producing a normal-shaped gland that was one-fifth the size of that in age-matched controls (Fig. 6G-I). 3D reconstruction showed that the hypoplastic thyroid in mutants had a normal anatomical shape (Fig. 6J). 3D reconstruction also revealed a smaller diameter of the trachea, which is expected based on the profound impairment of lung development in the absence of Fgf10 (Bellusci et al., 1997; Sekine et al., 1999). *Fgf10^−/−^* mice were generally growth-retarded but the weight of newborn mutant pups lacking limbs (0.88±0.03 g; *n*=3) was reduced by only 30% compared with wild-type siblings (1.24±0.05 g; *n*=7). These observations thus provided direct evidence that Fgf10 is a major and specific growth stimulus of the orthotopic thyroid gland before birth. Residual thyroid growth amounting to only 15% of the gland volume at E18.5 (Fig. 6I) can be attributed to Fgf10-independent mechanism(s).

### Fgf10 promotes branching growth of Sox9^+^ thyroid progenitors

Thyroid volume calculations further indicated that impaired growth in the absence of Fgf10 was relatively more pronounced between E12.5 and E15.5 than between E15.5 and E18.5 (Table 1). Accordingly, branching growth diminished leading to shortened epithelial cords with stubby endings (Fig. 6A,B). Surprisingly, the fraction of Ki-67^+^ epithelial cells did not significantly decrease until late thyroid development accompanying differentiation and folliculogenesis (Fig. 7A). Abundance of Ki-67^+^ cells were scattered throughout the thyroid rudiment rather than accumulated in the distal tip of branches (Fig. 7B,D,D′). Notably, the thyroid rudiment of *Fgf10^−/−^* mutants contained numerous Sox9^high^ cells that occasionally accumulated in immature buds (Fig. 7E) but more often were randomly dispersed throughout the parenchyma (Fig. 7E′). The relative number of Sox9^+^ cells did not differ much from that of the normal thyroid. Taken together, these observations indicated that Fgf10-stimulated embryonic thyroid growth cannot solely be explained by a mitogenic effect, i.e. a morphogenetic activity contributes, and that Fgf10 is not required for Sox9 expression in thyroid progenitors.

**Table 1.**
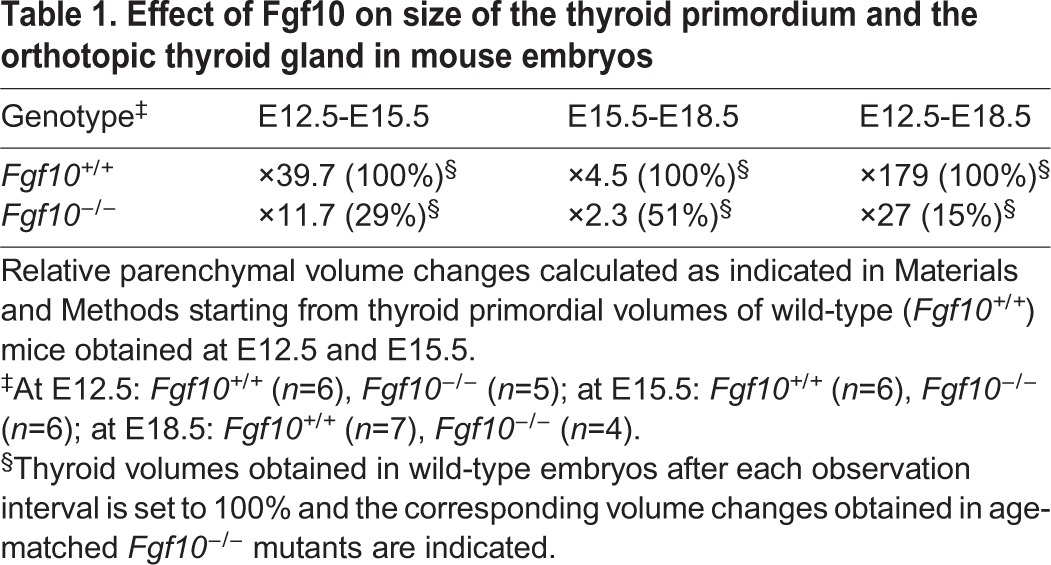
**Effect of Fgf10 on size of the thyroid primordium and the orthotopic thyroid gland in mouse embryos**

### Fgf10 stimulates thyroid follicular growth

**Fig. 8.**
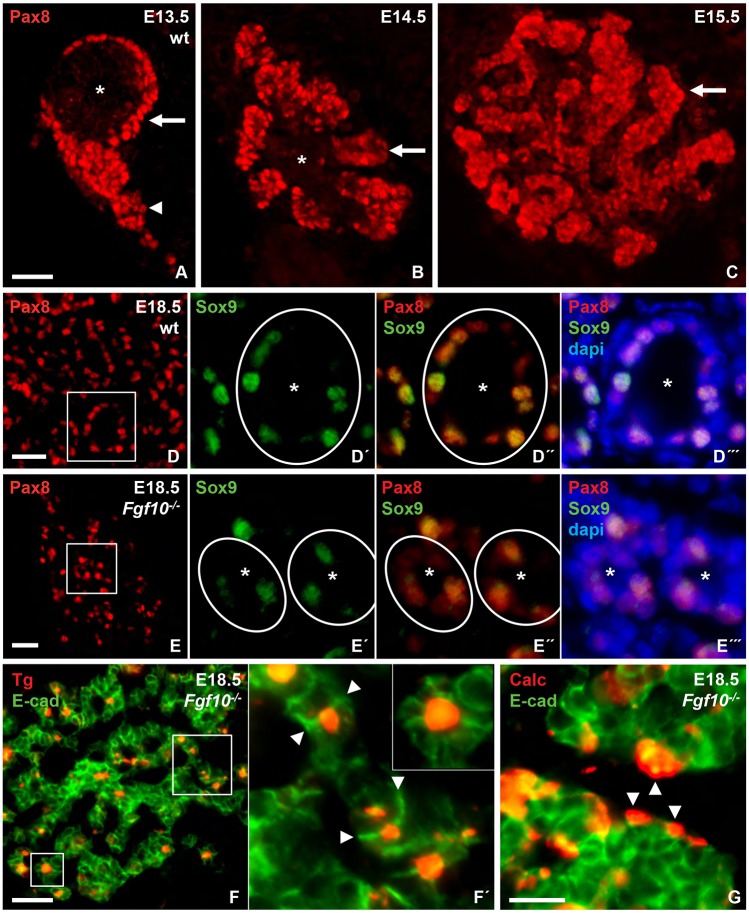
**Embryonic thyroid differentiation and folliculogenesis in *Fgf10*-deficient mice.** (A-E‴) Pax8 expression during thyroid growth (A-C) and differentiation (D-E‴). In A-C, arrows indicate branching growth progression, arrowhead indicates thyroid isthmus and asterisks indicate Pax8-negative ultimobranchial body remnant. D′-D‴ and E′-E‴ show Pax8/Sox9 co-expression (magnifications of the boxed areas in D,E); asterisks in D-E‴ indicate follicles. (F,F′) Thyroglobulin synthesis and follicle formation; F′ shows magnifications of the boxed areas in F showing bi- and multicellular microfollicles. Arrowheads indicate E-cadherin^+^ intercellular borders superimposing a lumen formed by two adjacent cells. (G) Thyroid C cells. Arrowheads indicate parafollicular localization. Calc, calcitonin, dapi, nuclear stain; Tg, thyroglobulin. Scale bars: 50 µm (A-E); 25 µm (F); 10 µm (F′,G).

Pax8, a key factor in thyroid differentiation (Nilsson and Fagman, 2017), was monitored to determine whether lack of Fgf10 affected the expression of Pax8 and its involvement in folliculogenesis. In normal thyroid development, Pax8 was found to be ubiquitously expressed in thyroid progenitors during branching growth (Fig. 8A-C). As cells reorganized into follicles Pax8 showed a more heterogeneous pattern (Fig. 8D) and was often co-expressed with Sox9 (Fig. 8D′-D‴). In *Fgf10* null embryos of the corresponding age (E18.5), the total number of Pax8^+^ thyroid cells was much reduced (Fig. 8E) and Pax8^+^/Sox9^+^ cells were in minority in follicle-like structures (Fig. 8E′-E‴). Notably, the parenchyma consisted of a reticular network in which microfollicles accumulating thyroglobulin was observed (Fig. 8F). Such follicles mostly consisted of only two adjacent cells present in rows in slender epithelial cords (Fig. 8F′) whereas multicellular follicles were less frequent (Fig. 8F′, inset). It was previously demonstrated that *de novo* follicle formation initially involves bicellular structures sharing a microlumen being established by coalescence of apical vesicles (Hilfer, 1979; Ishimura and Fujita, 1979; Nilsson and Fagman, 2017). These findings thus indicated that Fgf10 has no role in the differentiation of progenitors into polarized thyroid epithelial cells but is required for the enlargement and maturation of nascent follicles.

Cytodifferentiation of proximal airway progenitors involves replacement of Sox9 with Sox2, which also renders cells unresponsive to branch-inducing signals (Gontan et al., 2008). Fgf10 simultaneously represses Sox2 expression in Sox9^+^ lung progenitors as a means to prevent premature differentiation of the distal airways (Volckaert et al., 2013). The present findings in both normal and *Fgf10*-deficient embryos of no apparent proximal-distal boundaries of folliculogenesis and functional differentiation argue against a similar role of Sox9 in preserving the undifferentiated thyroid progenitor phenotype. As Sox2 is excluded from the thyroid domain of anterior endoderm (Ishii et al., 1998), we recombined *Sox2Cre* mice with the *mTmG* reporter to investigate whether thyroid progenitors might express Sox2 in later stages of thyroid development. This showed that Sox2 is entirely excluded from the thyroid follicular lineage until adulthood (Fig. S7). From this, it is evident that the proximal-distal Sox2/Sox9 patterning that characterizes the lung development and differentiation is not reproduced in the embryonic thyroid.

### Fgf10 controls formation of angiofollicular units

**Fig. 9.**
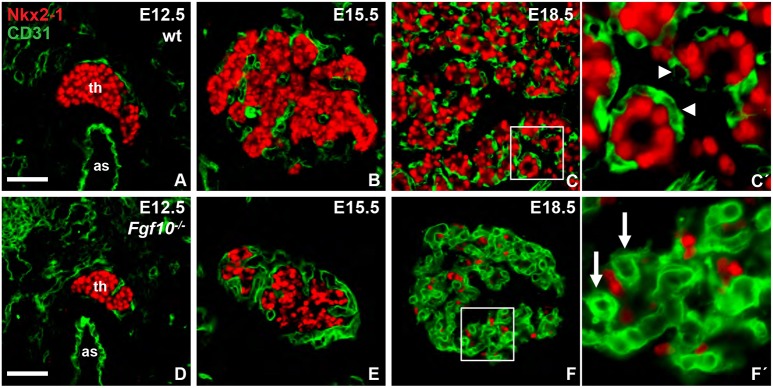
**Vascularization of embryonic thyroid in *Fgf10*-deficient mice.** (A,D) Thyroid primordium, midline sections. (B,E) Prospective thyroid lobe. (C,F) Thyroid lobe in late development. (C′,F′) C′ shows angiofollicular units (magnification of boxed area in C). Arrowheads indicate thyroid capillaries. F′ shows deranged capillary network (magnification of boxed area in F). Arrows indicate capillaries with abnormal size. Scale bars: 50 µm (in A for A-C; in D for D-F).

Recent observations indicate that proper follicle formation and maturation requires reciprocal interactions between thyroid progenitors and endothelial cells invading the embryonic thyroid (Hick et al., 2013; Villacorte et al., 2016). It was therefore of interest to investigate whether neovascularization of the gland gradually taking place during lobe growth (Fagman et al., 2006) might also be hampered in *Fgf10*-deficient mice. There were no differences in microvessel distribution between wild-type and mutant thyroids before lobe formation (Fig. 9A,D) and during branching morphogenesis (Fig. 9B,E), although the hypoplastic gland contained fewer capillary sprouts. This could correspond to the modestly reduced Ki-67 index obtained for thyroid mesenchyme in E15.5 mutant thyroids (Fig. 7C). Accompanying thyroid differentiation endothelial cells reorganized into a capillary network that enclosed the individual follicles forming so-called angiofollicular units (Fig. 9C,C′), which are crucial to establish and maintain thyroid endocrine function (Colin et al., 2013; Jang et al., 2017). Strikingly, at this developmental stage *Fgf10* mutants showed exaggerated microvessel formation that much exceeded the density of Nkx2-1^+^ parenchyma (Fig. 9F). Moreover, the vessel morphology differed in favor of tortuous capillaries of variable caliber and endothelial thickness that seemingly entrapped Nkx2-1^+^ cells in minority (Fig. 9F′). It is noteworthy that capillaries were sparse in the mesenchyme surrounding the thyroid rudiment in *Fgf10^−/−^* mice (Fig. 9E,F), indicating that the abundance of thyroid vasculature was probably elicited by thyroid progenitors themselves, which are known to secrete angiogenic factors (Hick et al., 2013), and not the result of general vessel abnormalities. Together, these findings suggest that Fgf10 is required for a coordinated and balanced proliferation and interaction of follicular and endothelial cell precursors as the embryonic thyroid attains its endocrine role.

It has previously been shown that propagation of the C cell lineage within the thyroid requires interplay with the follicular epithelium involving ephrin-Eph bidirectional signaling (Andersson et al., 2011). We therefore analyzed whether thyroid C cells might be affected by impaired growth of follicular cells in *Fgf10*-deficient embryos. However, C cells were found to be numerous and normally distributed close to the follicular epithelium also in the absence of Fgf10 (Fig. 8G). This indicates that Fgf10 is not involved in thyroid-UBB fusion or C cell differentiation. If anything, the follicular contribution to thyroid hypoplasia in *Fgf10* mutant mice might be underestimated by the C cell content.

## DISCUSSION

**Fig. 10.**
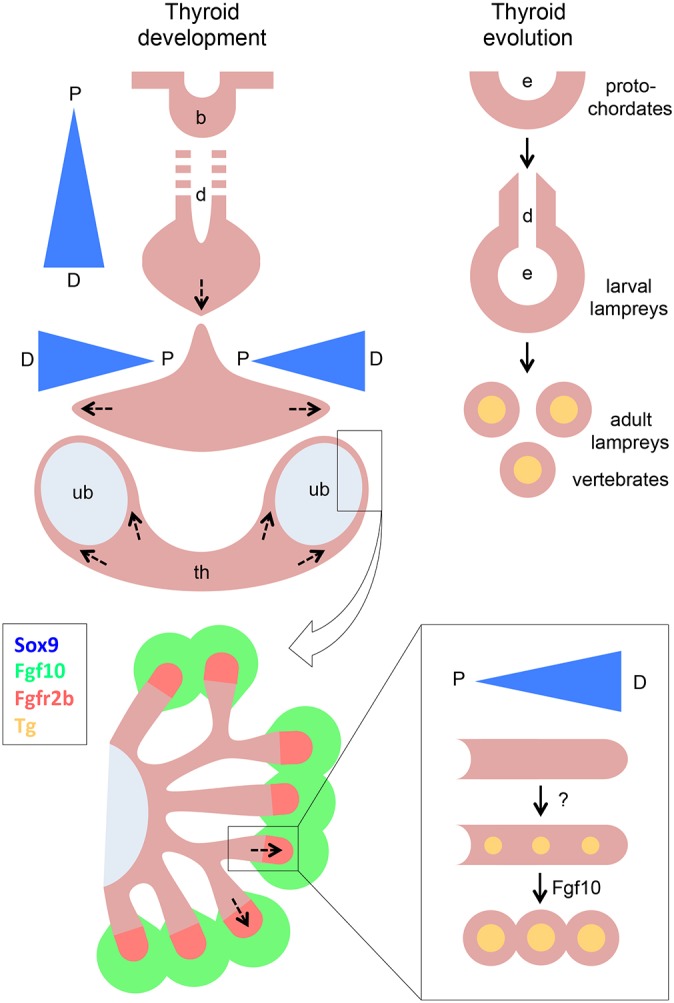
**Proposed models of branching morphogenesis in thyroid development.** Left: Bifurcation of the thyroid primordium into two lobes connected across the midline by the isthmus portion may be considered as the first generation of branching morphogenesis. Regression of the thyroglossal duct disconnects the embryonic thyroid from the pharyngeal endoderm. In late thyroid development, branching growth stimulated by Fgf10 derived from adjacent mesenchyme promotes the generation and maturation of follicles and acquisition of final organ size. Arrows indicate growth directions. The precise role of differentially expressed Sox9 in these processes, presumably acting in concert with other thyroid transcription factors (e.g. Nkx2-1 and Pax8), remains to be clarified. Right: An ancestral budding-branching mechanism could hypothetically be involved in an evolutionary switch of thyroid homologs from the exocrine endostyle found in protochordates to the follicular thyroid gland present in all vertebrates with the metamorphosing lamprey representing an intermediate species. b, bud; d, duct (thyroglossal); D, distal; e, endostyle; P, proximal; th, thyroid; ub, ultimobranchial body (the remnant after being fused with the midline thyroid primordium).

The present study identifies a novel mechanism of growth specific to branching morphogenesis in the embryonic thyroid in mice. The branching program is essentially launched in late thyroid development as the bilobed anatomy is being shaped prior to progenitor cell differentiation, and is stimulated by Fgf10 derived from the thyroid stroma (Fig. 10, left). Branching transforms the solid thyroid primordium into an arborized tissue that facilitates folliculogenesis synchronously in all parts of the gland. Fgf10-induced branching growth is responsible for more than 80% of thyroid enlargement before birth.

The rationale for adopting a branching type of morphogenesis is not obvious considering the endocrine function of the follicular thyroid lacking a ductal system for secretion. However, the exocrine features of the ancestral equivalent to the thyroid gland in invertebrates might provide an answer. In protochordates, such as *Ciona* and *Amphioxus*, a specialized filter-feeding domain of the ventral pharyngeal wall called the endostyle contains epithelial cells that metabolize iodine and secrete iodinated compounds into the alimentary canal (Gorbman, 1955; Nilsson and Fagman, 2017). In lampreys, an ancient lineage of vertebrates, the larval endostyle is converted upon metamorphosis into a follicular thyroid that is present only in the adult animal (Marine, 1913; Kluge et al., 2005). However, before this occurs the endostyle in lampreys forms a multi-chambered structure that communicates with the pharyngeal cavity by a slender duct reminiscent of an exocrine gland (Fig. 10, right) and which presumably is homologous to the thyroglossal duct in mammals (Kluge et al., 2005). Notably, the thyroglossal duct is a transient structure that connects the thyroid to the endoderm as the budding primordium moves downwards and bifurcates prior to lobulation (Fig. 10, left) (Nilsson and Fagman, 2017). A primitive or rudimentary branching morphogenesis program might thus be involved in a transitional phase in thyroid evolution from an exocrine to an endocrine gland. It is striking that thyroid follicular cells, unlike most if not all other endocrine cell types, show a strict apical-basal polarity typical of a simple epithelium and display exocrine features by secreting their main product thyroglobulin into the follicular lumen where it becomes iodinated (Nilsson and Fagman, 2017). From a phylogenetic viewpoint, conversion of an ancestral exocrine thyroid to an endocrine organ accompanied the evolution of terrestrial animals with increasing demands of thyroid hormone supply but in scarcity of iodine, which in marine environments is much more abundant. By this mechanism, exocrine secretion was retained but now routed into an enclosed compartment, the follicle lumen, serving as a site of thyroid hormone biogenesis and storage place of both prohormone and sequestered iodine. Branching growth of the embryonic thyroid gland is consistent with this hypothesis.

We found that absence of Fgf10 markedly reduced thyroid progenitor cell proliferation during branching growth. This conforms to previous observations in the developing lung (Bellusci et al., 1997; Sekine et al., 1999; Abler et al., 2009), exocrine pancreas (Bhushan et al., 2001; Hart et al., 2003; Norgaard et al., 2003; Dong et al., 2007; Manfroid et al., 2007; Kobberup et al., 2010) and salivary gland (Jaskoll et al., 2005; Steinberg et al., 2005), which collectively show that Fgf10 stimulates ductal organogenesis by promoting epithelial cell multiplication leading to branch elongation. However, besides being a mitogen, Fgf10 might promote developmental growth by additional mechanisms, for example, in the developing lung *Fgf10* does not seem to directly stimulate cell proliferation (this function is manufactured by Fgf7) but rather promote cell migration with impact on bud elongation and branching (Francavilla et al., 2013). We observed in mutant thyroids stubby and shortened parenchymal cords, and although many cells were Ki-67 positive they did not accumulate in those rudimentary branches. Moreover, in the absence of Fgf10 the relative decrease in Ki-67 cell numbers was less pronounced in branching parenchyma than following differentiation of the gland despite the fact that both the natural growth rate and inhibition of growth (in the absence of *Fgf10*) peaked during branching morphogenesis. Together, this suggests that Fgf10-dependent embryonic thyroid growth involves a dual mechanism, which on one hand promotes the generation of cells by a direct mitogenic effect, acting on both progenitors and follicles, and on the other hand stimulates branching morphogenesis that indirectly promotes progenitor cell proliferation.

In lung development, Fgf10 promotes self-renewal of distal airway progenitors by preventing their premature differentiation to specialized bronchial and alveolar phenotypes (Volckaert et al., 2013). In this process, Sox9 seems to play a crucial role by executing the branching program downstream of Fgf receptor activation (Chang et al., 2013). In pancreas morphogenesis, a feed-forward mechanism operates by which mesenchymal Fgf10 upregulates Sox9, which in turn promotes expression of Fgfr2b required for maintenance of the pancreatic progenitor cell pool (Seymour et al., 2007, 2012). More recently, in the salivary gland Sox9 was found to exert an additional function prior to branching by establishing the identity of distal progenitors without which branching fails (Chatzeli et al., 2017). In the present study, we found that Sox9 is neosynthesized and enriched in the primary thyroid bud and, later on, is upregulated in thyroid progenitors as these cells were about to hedge in the UBBs and cluster to form a handful of discrete buds, seemingly with the UBB rudiment as a proximal support, from which branching growth continued (Fig. 10, left). Sox9 expression in embryonic thyroid thus shows conspicuous similarities to the expression pattern of Sox9 in primordial tissues of exocrine glands during onset of branching morphogenesis prior to the formation of ducts (Thomsen et al., 2008; Chatzeli et al., 2017), with the exception that Sox9 in thyroid progenitors does not seem to be mastered by the Fgf10-Fgfr2b pathway.

Unfortunately, attempts to conditionally delete *Sox9* by employing the *Nkx2-1* promoter as Cre driver were inconclusive. After recombination, loss of Sox9 expression was evident in many but far from all cells in the embryonic thyroid, suggesting that incomplete inactivation at a critical stage might not be sufficient to reveal loss of function in *Nkx2-1Cre;Sox9^fl/fl^* embryos. Indeed, a mixed phenotype was previously observed in lung development after mosaic deletion of *Sox9* using a similar *Nkx2-1^Cre^* allele (Chang et al., 2013). Functional redundancy, evident among the three members of the SoxE gene family (Stolt et al., 2004; Barrionuevo and Scherer, 2010), might also explain the lack of effect of the single gene knockout. At present it is thus not possible to resolve the exact role(s) of Sox9 and its putative transcriptional partners and targets in thyroid development. Nonetheless, the fact that nuclear accumulation of Sox9 in the thyroid bud coincides spatiotemporally with activation of a network of thyroid transcription factors, including Nkx2-1 and Pax8, which before budding act independently of each other (Parlato et al., 2004), is intriguing and suggests that Sox9 might be involved in this regulatory switch.

Another interesting aspect is the lack of Sox9 expression in endocrine organs except the thyroid (The Human Protein Atlas; Uhlen et al., 2015), which was confirmed in the present study. In the embryonic pancreas, which develops into a mixed exocrine-endocrine tissue, Sox9 is ubiquitously expressed in multipotent progenitors but downregulated as endocrine islet cells differentiate whereas Sox9^+^ progenitors continue to contribute to the exocrine pancreas both developmentally and in adulthood (Furuyama et al., 2011). A preserved Sox9 expression in the thyroid follicular lineage but not in the parathyroid and UBB/C cell lineages, as observed in the present study, supports the hypothesis that the thyroid gland evolved from an exocrine ancestor, and provides a rationale for thyroid follicular cells to retain fundamental exocrine features (i.e. exocytosis of thyroglobulin to the follicle lumen).

Data presented in this study demonstrating lack of an ISH signal for *Fgf10* and essentially no effect of *Fgf10* knockout in the thyroid primordium before lobe formation indicate that the burst of progenitor cell proliferation taking place earlier on in thyroid development (Nilsson and Fagman, 2017) is Fgf10 independent. The fact that *Fgfr2b* is already expressed at this developmental stage favors a role of other Fgfs targeting the same receptor. For example, Fgf7 has been shown to induce epithelial budding and initial bud growth in both lung and salivary gland (Bellusci et al., 1997; Steinberg et al., 2005; Francavilla et al., 2013). So far, no functional studies specifically on Fgf7 and thyroid have been conducted, and Fgf7 also appears to be expressed in the embryonic thyroid at a later stage coinciding with lobe formation and growth (Finch et al., 1995). However, involvement of multiple Fgfs in early thyroid development is suggested from reports of thyroid agenesis in mouse mutants expressing a soluble dominant-negative Fgfr (Celli et al., 1998) or deficient in *Fgfr2IIIb* (Revest et al., 2001). Disruption of the *fgf8* gene in zebrafish results in both reduced size of the thyroid primordium and diminished number of follicles (Wendl et al., 2007). In mice, deletion of *Fgf8* also leads to a smaller thyroid primordium involving a paracrine loop conducted by Tbx1 expressed in adjacent mesoderm (Lania et al., 2009). It is envisaged that lack of Fgf8 reduces the number of progenitors committed to a thyroid fate, which might at least partly explain thyroid dysgenesis in the absence of Tbx1 (Fagman et al., 2007). However, the *Tbx1* mutant thyroid phenotype is confined to a hypoplastic rudiment in the midline indicating that loss of growth commences before bilobation takes place. From this, it can be concluded that any complementary growth factors to Fgf10 operating in late thyroid development and that may contribute to final organ size remain to be identified.

A recent study reported a unilateral thyroid remnant in *Fgf10^−/−^* embryos (Teshima et al., 2016). We did not observe thyroid hemiagenesis in our cohort of *Fgf10* null mice, which consistently displayed symmetric hypoplasia of the otherwise normal-shaped gland. Differences in genetic background could explain phenotypic variations of thyroid dysgenesis although the pathogenetic mutations are identical as in *Nkx2-1*/*Pax8* compound heterozygous mice (Amendola et al., 2005, 2010). Teshima et al. (2016) also found that conditional inactivation of Fgf10 in neural crest (*Wnt1Cre;Fgf10^fl/fl^* mutants) reduced thyroid size although to a lesser degree than after global inactivation, suggesting that mesodermal Fgf10 also likely contributes to thyroid development. Nonetheless, this study and ours localized Fgf10 to crest-derived mesenchyme that invests the thyroid primordium, which provides mechanistic insight to thyroid developmental defects reported to occur after neural crest ablation (Bockman and Kirby, 1984; Kameda et al., 2013; Maeda et al., 2016). It is important to note that the phenotype of *Fgf10* null mutant mice does not comprise thyroid agenesis, which was originally reported (Ohuchi et al., 2000) and thus erroneously stated in a recent review paper on the multifunctional role of Fgf10 in development (Itoh, 2016).

Growth regulation of the embryonic thyroid differs greatly from that of the adult gland; the multiplication rate is highest in fetal thyroid cells whereas the mitotic activity diminishes in childhood and is nearly undetectable in adult thyrocytes, which do not normally regenerate (Coclet et al., 1989; Saad et al., 2006). Enhanced thyroid growth *in utero* and infancy is likely to confer a high susceptibility to mutagens and an increased risk of developing childhood thyroid cancer (Williams, 2015). Unlike in adult thyroid cells, thyroid-stimulating hormone (TSH) from the pituitary has no role in embryonic thyroid growth (Hilfer and Searls, 1980; Postiglione et al., 2002). In fact, thyroid progenitor cells are intrinsically resistant to TSH receptor activation (Postiglione et al., 2002). With this prospect in mind, it is reasonable to assume that Fgf10-mediated growth predominantly in the developing thyroid could be of relevance also to thyroid cancer development. The present study also provides a novel pathogenetic mechanism for severe hypoplasia of the in-place thyroid gland. In humans, disruption of FGF10-FGFR2IIIb signaling causes a range of complex developmental disorders including cleft lip/palate (Rice et al., 2004), autosomal dominant aplasia of lacrimal and salivary glands (ALSG) (Entesarian et al., 2005) and lacrimo-auriculo-dento-digital (LADD) syndrome (Milunsky et al., 2006; Riley et al., 2007). To date, there are no reports linking thyroid dysgenesis, the most common cause of congenital hypothyroidism, to *FGF10* mutations. However, in view of the fact that for most patients with thyroid dysgenesis the causal mechanism is unknown (Van Vliet, 2003), FGF10 and other factors associated with this signaling pathway are potentially new candidate genes worth exploring.

## MATERIALS AND METHODS

### Animals

Targeted disruption of *Fgf10* and PCR genotyping of genomic DNA have been previously described (Sekine et al., 1999). *Fgf10^+/−^* mice maintained on a C57BL/6 background were bred to generate *Fgf10* null heterozygous and homozygous mutant embryos and wild-type siblings that were collected at E9.5, E12.5, E15.5 and E18.5. *Nkx2-1Cre*, *Wnt1Cre*, *Sox2Cre*, *Sox9^flox^* and *ROSA^mTmG^* (hereafter called *mTmG*) mice were obtained from Jackson Laboratory and maintained on a C57BL/6 background. *Nkx2-1-Cre* were crossed with *Sox9^flox^* and *mTmG* to generate *Nkx2-1Cre;Sox9^fl/+^* and *Nkx2-1Cre;Sox9^fl/fl^* and *Nkx2-1Cre;mTmG* embryos and pups, respectively. Yolk sac or embryonic tails were used for DNA extraction and PCR genotyping. Three or more independent experiments on embryos obtained from different litters were analyzed for each genotype and developmental stage. AKM6 at the Clinical Research Center, Karolinska University Hospital (Huddinge, Sweden) was employed for re-derivation of *Fgf10^+/−^* mice before transfer to the local animal facility. The local ethics committee at the University of Gothenburg approved all experiments.

### Immunoreagents

Primary antibodies used for immunofluorescence were: rabbit anti-Nkx2-1 (1:1000; PA0100, EAtlab srls); rabbit anti-Fgf10 (1:500; ABN44, EMD Millipore); rabbit anti-Fgfr2 (1:5000; ab10648, Abcam); goat anti-hSOX9 (1:500; AF3075, R&D Systems); rat anti-E-cadherin (ECCD-2; 1:500; 205604, Calbiochem); rabbit anti-Ki-67 (1:100; ab15580, Abcam); rabbit anti-Pax8 (kindly provided by Roberto di Lauro, Universita di Napoli Federico II, Naples, Italy); rabbit anti-Tg (1:3000; A0251, Dako); rabbit anti-calcitonin (1:500; A0576, Dako). Secondary antibodies used include: Rhodamine Red-X-conjugated donkey anti-rabbit IgG (Jackson ImmunoResearch Laboratories); Rhodamine Red-X-conjugated donkey anti-goat IgG (Jackson ImmunoResearch Laboratories); biotin-conjugated donkey anti-rat IgG (Jackson ImmunoResearch Laboratories) and biotin-conjugated donkey anti-goat IgG (Jackson ImmunoResearch Laboratories) followed by Streptavidin-FITC (Dako).

### Immunostaining and fluorescence microscopy

Embryos were fixed with 4% paraformaldehyde overnight at 4°C and washed three times in PBS (pH 7.3) before cryoprotection in 30% sucrose overnight at 4°C, embedding in Tissue Tek compound (Sakura, Zoeterwoude, The Netherlands) and freezing at −80°C for storage. Serial sections (thickness 10 µm, sagittal for E9.5 and E12.5 and transversal for E15.5 and E18.5) were cut on a cryostat and collected on Superfrost Plus glass slides (Mentzel Gläser, Germany). Sections were permeabilized with 0.1% Triton X-100 in PBS for 20 min and blocked with 2% normal donkey serum (Jackson ImmunoResearch Laboratories) in PBS for 1 h before incubation with primary antibodies (listed above) diluted in blocking buffer overnight at 4°C. Secondary antibodies in blocking buffer were added for 60 min followed by incubation with streptavidin-FITC for 30 min, both at room temperature. Sections were counterstained with DAPI (Sigma-Aldrich) and finally mounted with Fluorescence Mounting Medium (Dako). Each incubation step was ended with washing 3×5 min with 0.1% Triton X-100 in PBS. Images were captured using a Zeiss Axioscope 2 Plus fluorescence microscope equipped with a Nikon DS-Qi1Mc camera and processed with NIS Element Imaging and ImageJ software.

### *In situ* hybridization (ISH)

Wild-type (wt) embryos were fixed, cryoprotected, embedded and cryosectioned as described above. Riboprobes were synthesized from plasmids containing rat Nkx2-1 (kindly provided by Luca Parrillo, Universita di Napoli Federico II, Naples, Italy), Fgf10 and FGFR2b cDNA (kindly provided by Amel Gritli-Linde, University of Gothenburg, Göteborg, Sweden). Digoxigenin-UTP labeling and *in vitro* transcription of plasmid cDNA into antisense and sense riboprobes were performed using the DIG RNA Labeling Kit (Roche Diagnostics) following to the manufacturer's instructions. The protocol employed for ISH was adapted from: ‘Digoxigenin-labeled *In situ* Hybridization for P1 mouse kidney sections’, available at http://www.gudmap.org/Research/Protocols/McMahon.html. In brief, embryo sections were air-dried for at least 1 h before fixed with 4% paraformaldehyde, treated with proteinase K and fixed again. Following acetylation, air-dried sections were incubated with 500 ng/ml anti-sense or sense riboprobes overnight at 68°C. Labeled sections were treated with RNase A (Roche Diagnostics), incubated overnight at 4°C with anti-digoxigenin antibody (Roche Diagnostics), and eventually with BM Purple AP Substrate (Roche Diagnostics) at room temperature for various time intervals, ranging from overnight to several days, to optimize specific labeling. Color development was stopped by formaldehyde fixation after which sections were air-dried and mounted with glycergel mounting medium (Dako). Stringent washing followed each ISH incubation step. Images were captured using a Nikon Eclipse E1000M microscope equipped with a Jenoptik ProgRes C7 camera and processed with Adobe Photoshop. No signals beyond background were detected with sense riboprobes (data not shown).

### Morphometry

Serial sections subjected to Nkx2-1 and E-cadherin (cadherin 1) co-immunostaining and encompassing the entire thyroid placode (at E9.5), primordium (at E12.5) or embryonic gland (at E15.5 and E18.5) were used for evaluation of thyroid size. Images were captured in a Zeiss Axioscope 2 Plus fluorescence microscope equipped with a Nikon DS-Qi1Mc camera and processed with NIS Element Imaging software. For E9.5, all Nkx2-1-positive cells of the thyroid placode were counted. For E12.5, as there were few other cell types in the primordium its perimeter was encircled in each section and the area calculated. For E15.5 and E18.5, owing to the greater organ size and considerable contribution of the stromal compartment only Nkx2-1/E-cadherin-positive parenchyma in every fourth section was encircled for area calculations. Data obtained from E12.5-E18.5 were then used for 3D reconstructions using WinSURF software version 4.3, which allows the relative volumes of objects to be determined and compared. For E18.5, the parathyroids and trachea were also reconstructed based on E-cadherin staining in the same sections.

### Statistics

Each data point shown in graphs represents an individual embryo and bars indicate the mean. For comparison of cell numbers (Nkx2-1^+^; Ki-67^+^), statistical analysis was performed using Student's *t-*test with a *P*-value <0.05 considered to be significant. For estimation of thyroid size changes, the mean volume of mutant glands were calculated as a percentage of the mean control volume of wt glands. Statistical analysis of thyroid volume estimates was determined using Student's *t*-test with *P*≤0.05 considered to be significant. All quantitative data and statistical analyses were assembled using GraphPad Prism version 6 (GraphPad Software).

## Supplementary Material

Supplementary information
