# Supplementary material for: A branching morphogenesis program governs embryonic growth of the thyroid gland
Source: Development. 2018 Jan 15;145(2):dev146829. doi: 10.1242/dev.146829 (PMC5825846; doi:10.1242/dev.146829)
Supplement: Supplementary information [file develop-145-146829-s1.pdf]

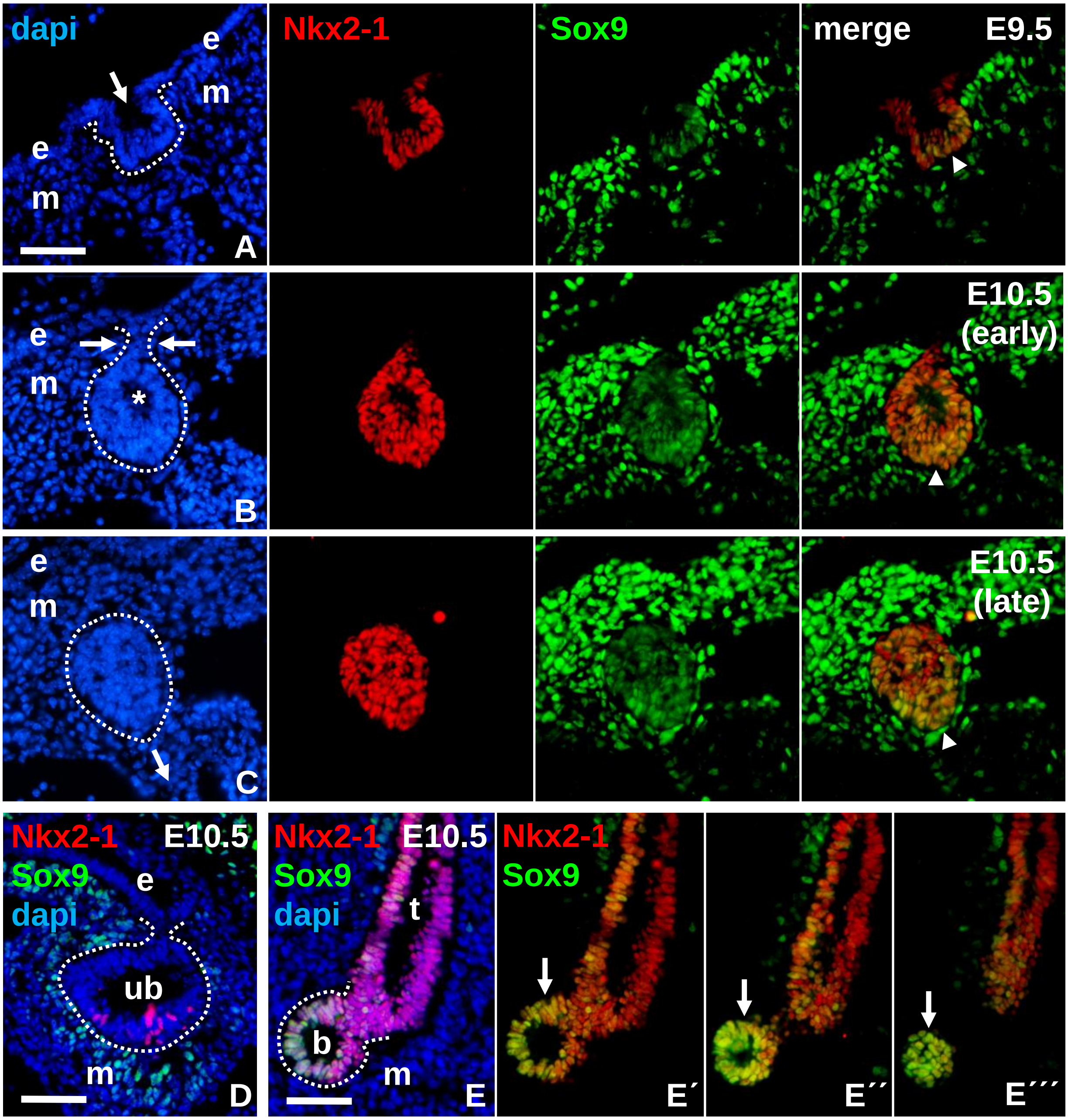

Fig. S1. Sox9 expression during organ budding from anterior endoderm. (A-C) Thyroid bud development involving invagination (A), budding (B) and detachment (C) from pharyngeal endoderm. The thyroid primordium is outlined in left panels stained with dapi. Asterisk indicates preserved lumen in bud. Arrows indicate direction of tissue movements. Arrowheads indicate distal portion of thyroid primordium in which Sox9+ progenitors accumulate. Sox9+ mesenchymal cells accumulate between thyroid bud and endoderm. (D) Formation of ultimobranchial body from the fourth pharyngeal pouch surrounded by Sox9+ mesenchyme. Epithelial cells lacking Sox9 start to express Nkx2-1. (E-E´´´). Distal Nkx2-1+ lung progenitors strongly express Sox9. Arrows in E´ series indicate distal branch captured in parallel sections. e, endoderm; m, mesenchyme; ub, ultimobranchial body; t, trachea (prospective); b, bronchial bud. Scale bars: 25 μm.

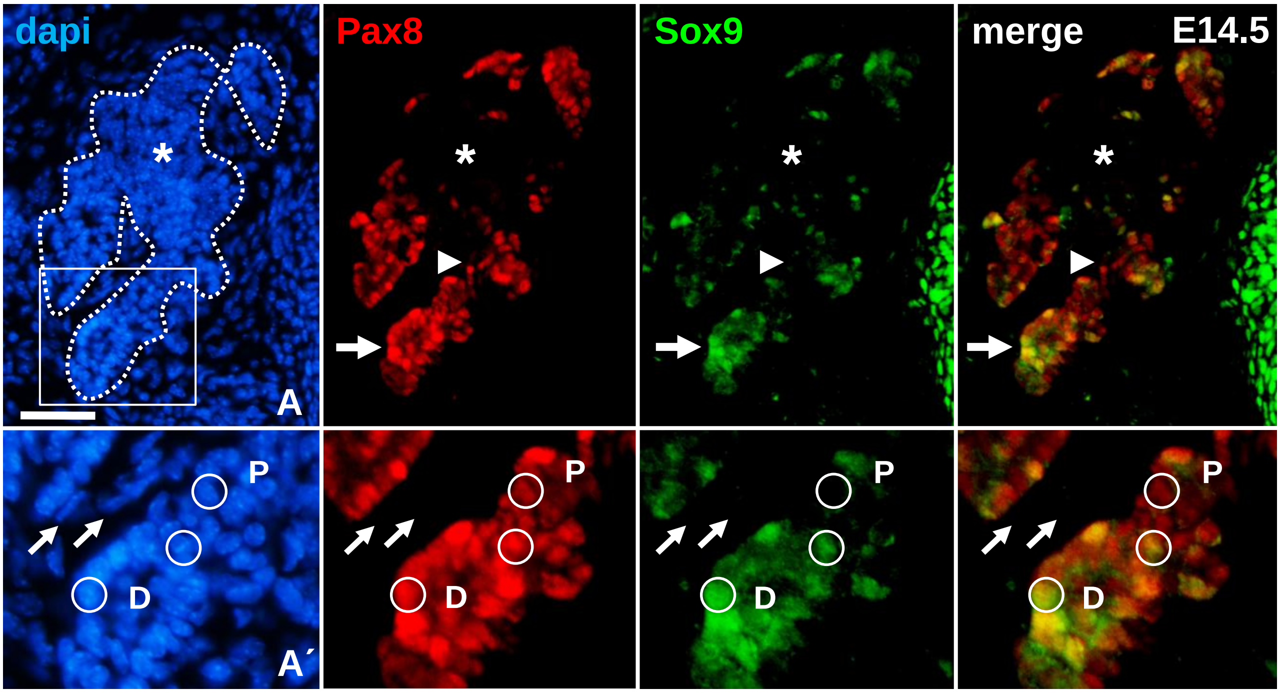

Fig. S2. Sox9 expression during thyroid branching morphogenesis. (A) Branching growth of thyroid progenitors is evident after the midline primordium is merging bilaterally with the ultimobranchial body. The outer border of the prospective thyroid lobe is outlined (dotted line in left panel) and the centrally located Sox9 negative ultimobranchial body remnant is indicated with asterisks. Thyroid follicular progenitors exclusively express Pax8. Sox9+ cells are preferentially located in the distal portion (arrow) whereas Sox9 is down-regulated in the proximal portion (arrowhead) of Pax8+ epithelial cords. (A´) Close-up of inset in (A) detailing the proximal-distal expression pattern of Sox9 in Pax8+ thyroid progenitor cells; some nuclei are encircled to highlight Sox9+^{high}, Sox9+^{low} and Sox9– cells, respectively. Small arrows indicate mesenchymal cells present in the space between parenchymal cords. P, proximal; D, distal. Scale bar: 25 µm.

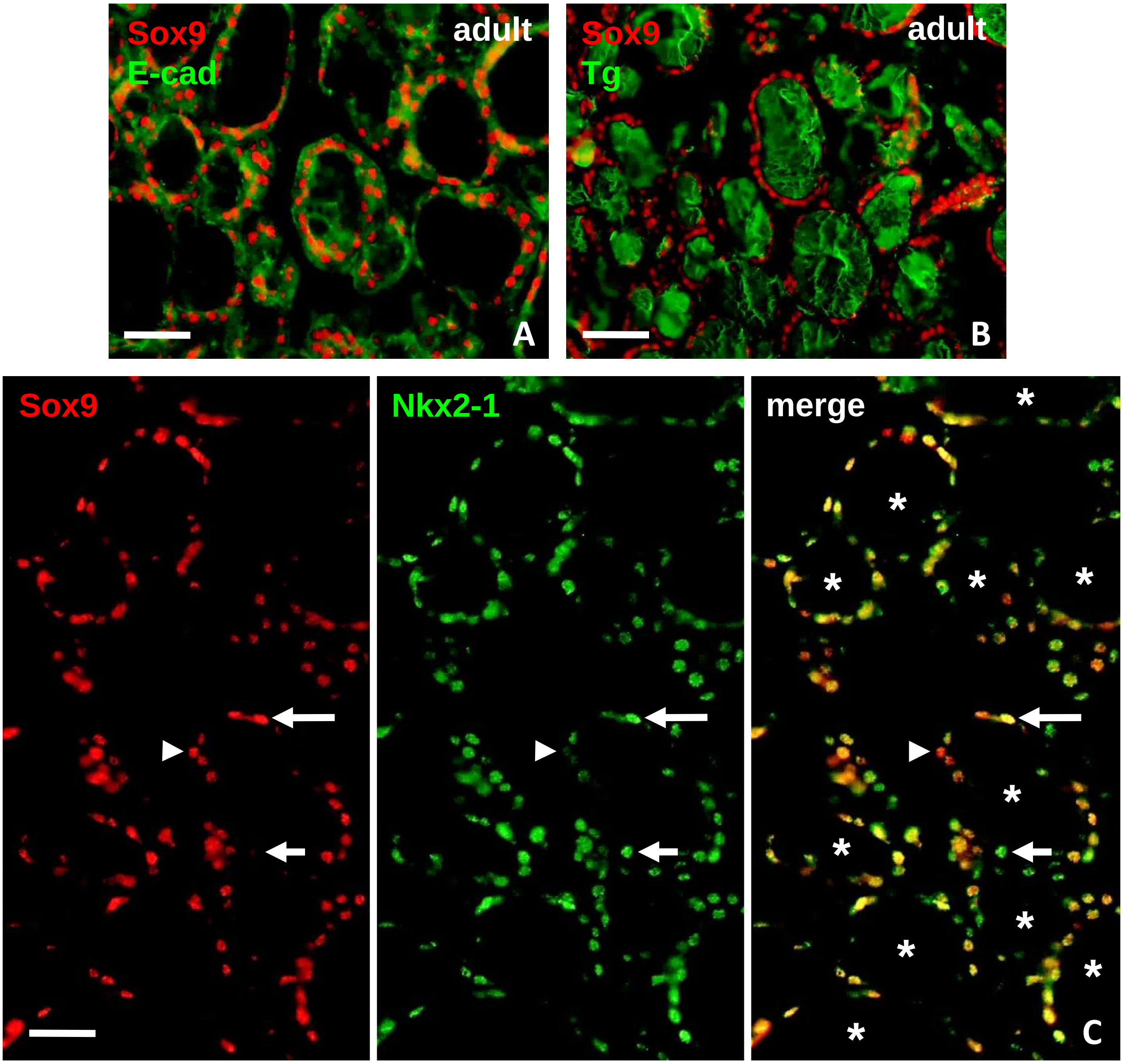

Fig. S3. Sox9 expression in adult mouse thyroid cells. (A, B) Sox9 expression is confined to the thyroid follicular epithelium expressing E-cadherin (A) and delimiting the follicular lumen filled with thyroglobulin-containing colloid (B). (C) Sox9 co-localizes with Nkx2-1 in the nuclei of thyrocytes. Notably, the relative expression levels of Sox9 and Nkx2-1 differ among cells comprising Sox9^{high}/Nkx2-1^{low} (arrowhead), Sox9^{high}/Nkx2-1^{high} (arrow) and Sox9^{low}/Nkx2-1^{high} (small arrow) profiles. Whether differential expression of Sox9 and Nkx2-1 reflects functional differences among cells within the same follicles (indicated by asterisks) is presently unknown. Scale bars: 50 μm.

Development • Supplementary information

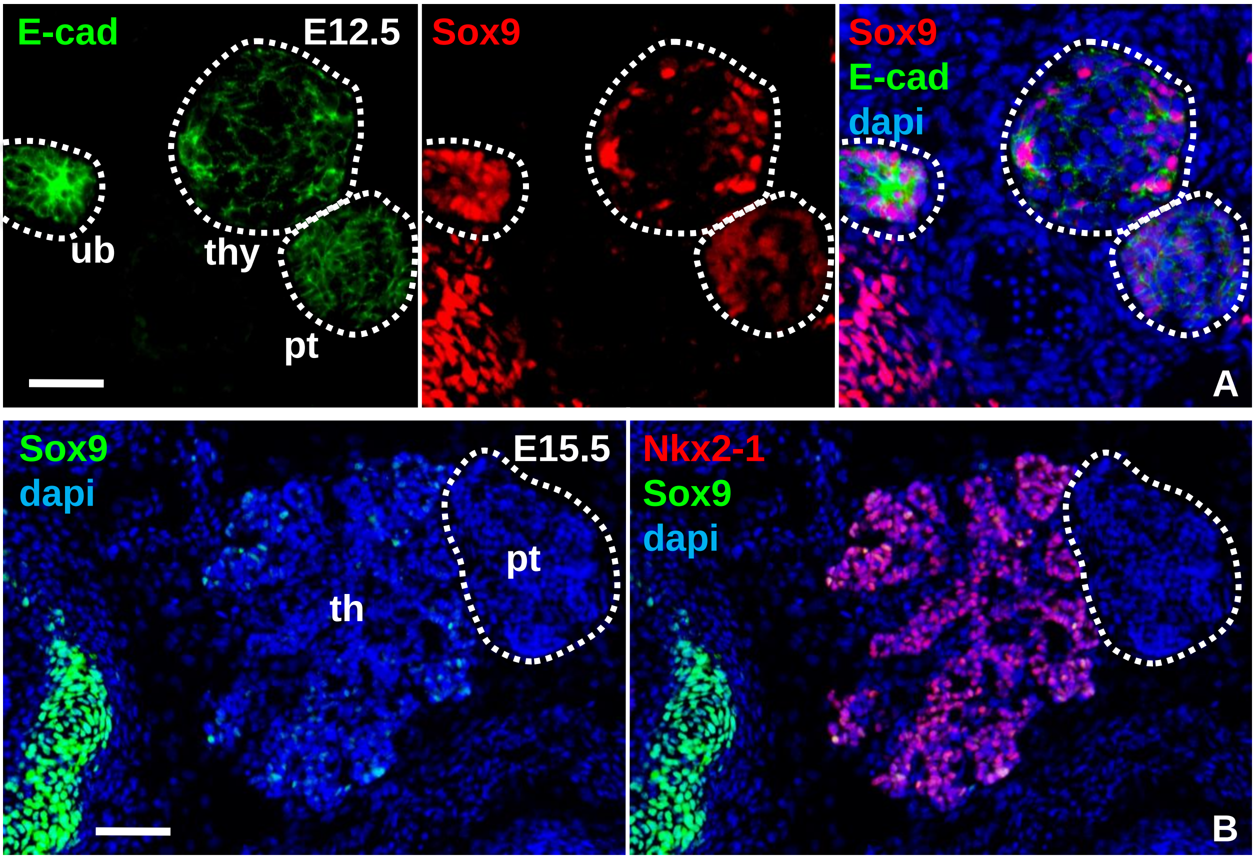

Fig. S4. Sox9 expression in pharyngeal pouch derivatives. (A) The left ultimobranchial body (ub) and rudiments of the thymus (thy) and the parathyroid gland (pt) identified by E-cadherin (E-cad) expression exhibit Sox9+ cells as these primordia (encircled) are detached from their respective pharyngeal pouch origins. Nearness of thymus and parathyroid reflects they develop from endoderm anlagen present in the same pouch. (B). Sox9 negative parathyroid gland (pt; encircled) located close the left thyroid lobe (th). Note distal Sox9+ cells in branching parenchyma of the thyroid. Scale bars: 50 μm (A), 100 μm (B).

Development • Supplementary information

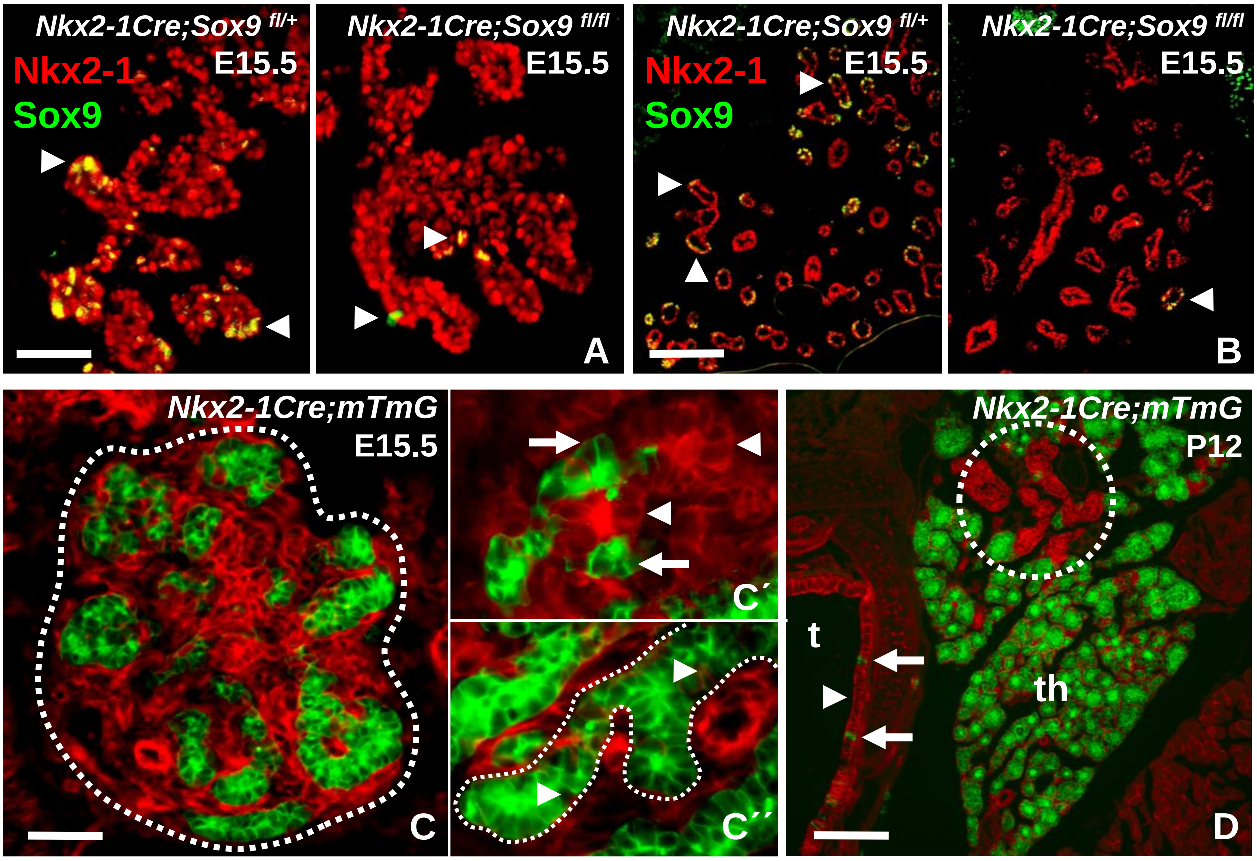

Fig. S5. Targeted knockout of *Sox9* in embryonic thyroid and lung cells with *Nkx2-1Cre*. (A, B) Immunofluorescence of Nkx2-1 and Sox9 in embryonic thyroid (A) and lung (B) in *Nkx2-1Cre;Sox9*$^{fl/+}$ and *Nkx2-1Cre;Sox9*$^{fl/fl}$ mouse E15.5 embryos. Arrowheads indicate preserved Sox9 expression in branching tips in *Sox9*$^{fl/+}$ mice (left images) and residual Sox9 expression in few parenchymal cells in *Sox9*$^{fl/fl}$ mice (right images). (C, D) Lineage tracing of *Nkx2-1Cre* mice crossed with the *mTmG* reporter. As shown in images, in this line progeny of Cre expressing cells will be inevitably green fluorescent (mG+) whereas cells in which Cre is not yet expressed or activated remain red fluorescent (mT+) indicating mosaicism. Thus, any floxed transgene that is crossed with *Nkx2-1Cre* will not be expressed by all Nkx2-1+ cells.(C) Prospective thyroid lobe at E15.5 consisting of both mG+ and mT+ parenchymal cells. (C ´) Segmentation of thyroid parenchymal cord into mG+ (arrows) and mT+ (arrowheads) portions at E15.5. (C ´ ´) Branching morphogenesis of mG+ thyroid progenitors (outlined). Note presence of sparse mT+ cells within the mG+ branch (arrowheads). (D) Signs of preserved mosaicism postnatally wih foci of mT expressing cells (encircled) in the left thyroid lobe (th) at P12. Note the respiratory epithelium of the trachea (t) is mainly mT+ (arrowhead) and exhibits only few mG+ cells (arrows). The poor EGFP labling as compared to thyroid and distal lung cells (not shown) is conceivably explained by the fact that Nkx2-1, although being ubiquitously expressed in the lung bud, is rapidly downregulated during branching morphogenesis thus limiting the possibility of expressing Cre in proximal airway cells. Scale bars: 25 µm (A), 200 µm (B), 50 µm (C), 200 µm (D).

Development • Supplementary information

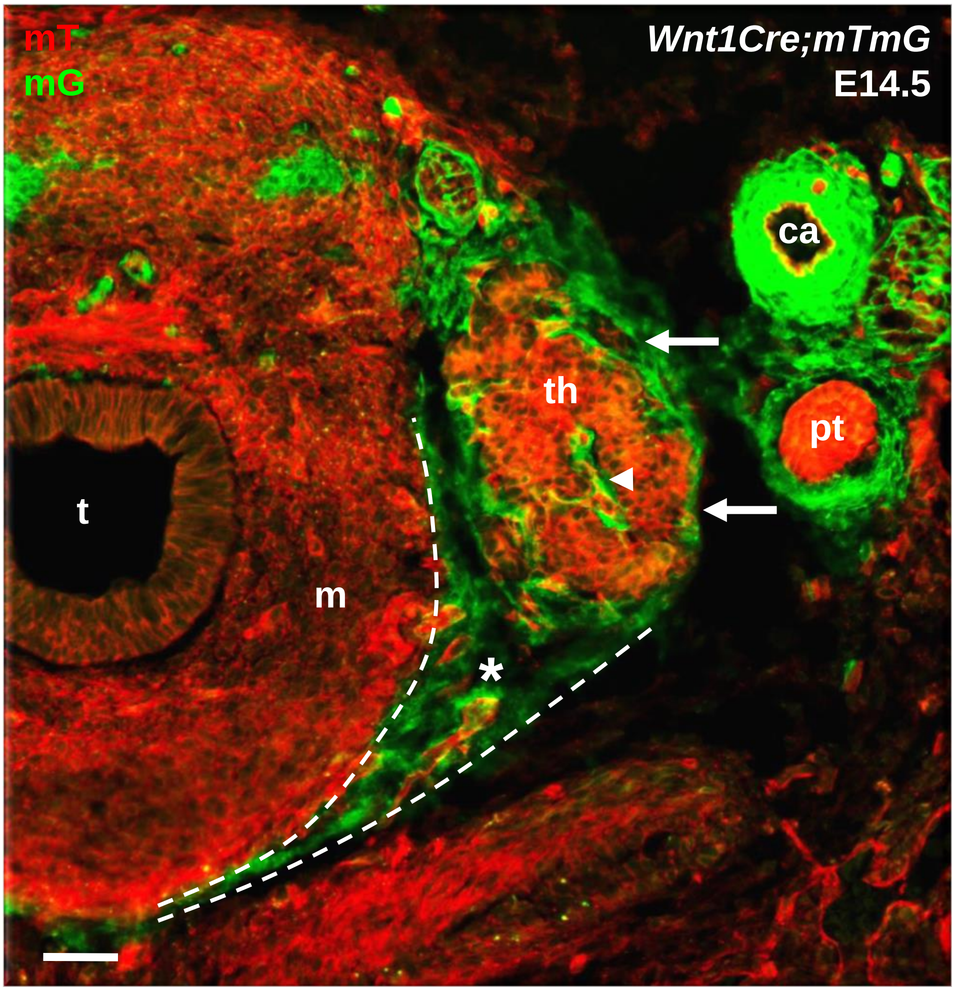

Fig. S6. Lineage tracing of Wnt1⁺ progeny in mouse thyroid development. *Wnt1Cre* mice were crossed with the *mTmG* reporter to pinpoint the contribution of neural crest-derived mesenchyme in thyroid organogenesis. It is previously known that Wnt1Cre faithfully labels the progeny of cranial neural crest during mouse embryogenesis (Chai et al., 2000). As shown in graph, mG+ cells invest the prospective thyroid lobe (th) at E14.5. Notably, the mG+ thyroid capsule and the associated mG+ domain (asterisk) extending ventromedially in front of the trachea (outlined) have the same distribution as *Fgf10* expressing mesenchyme (see Fig. 4E) suggesting they are identical. The mT+ collar of mesenchyme (m) surrounding the trachea (t) does not originate from neural crest. pt, parathyroid; ca, carotid artery. Scale bar: 25 µm.

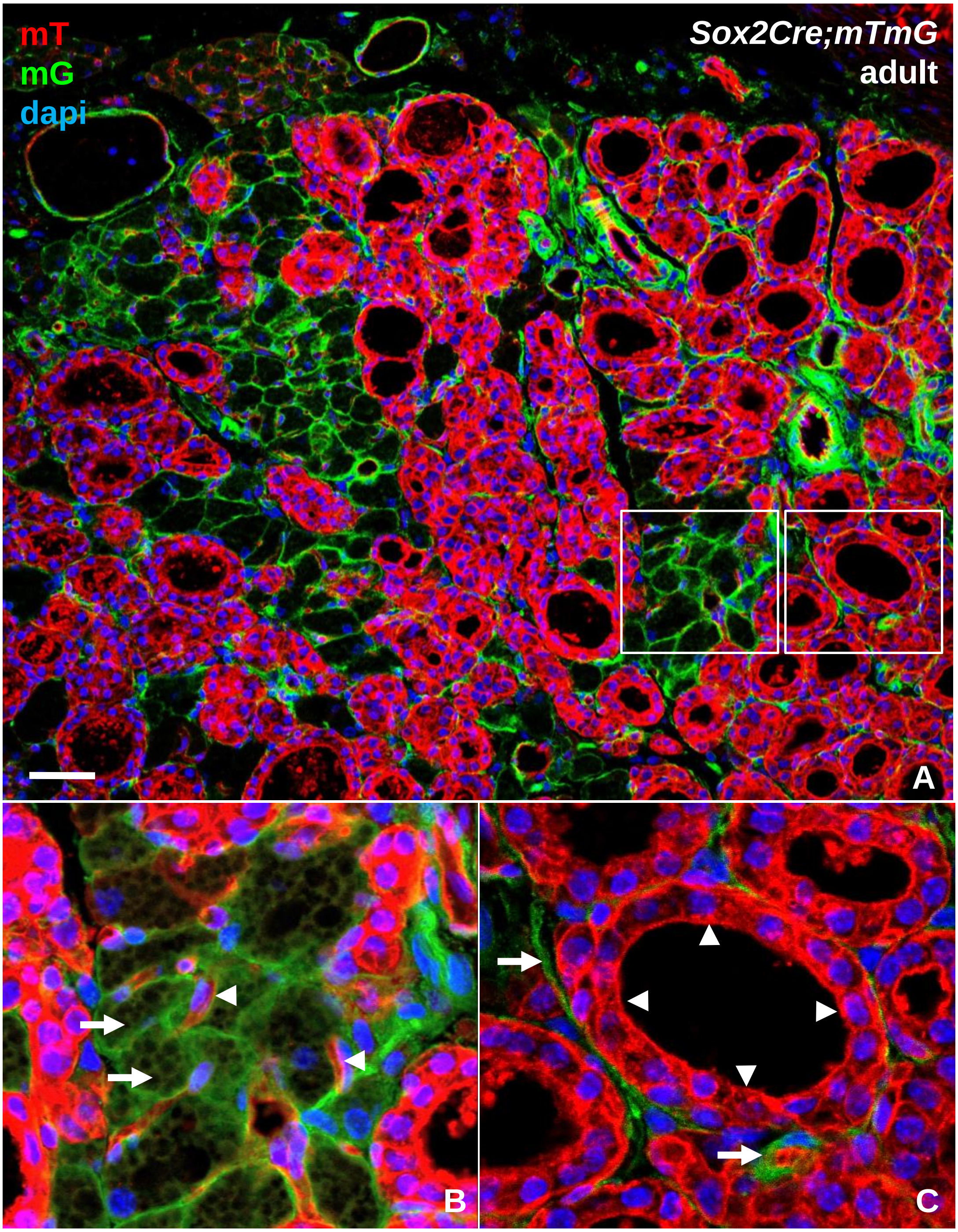

Fig. S7. Lineage tracing of Sox2+ progeny in mouse thyroid development. *Sox2Cre* mice were crossed with the *mTmG* reporter to investigate whether Sox2 is at all expressed in thyroid follicular cells. As shown in A, mG+ cells are limited to vessels and stroma whereas follicular cells remain mT+. This indicates that Sox2 is not expressed in thyroid progenitors at any developmental stage. Insets in A detail the Sox2+ progeny comprising thyroidal brown fat cells (arrows in B) and microvessels (arrows in C). Arrowheads in B and C indicate that all follicular cells and some stromal cells express mTomato. Scale bar: 25 µm.
